# Short-Term Starvation of Immune Deficient *Drosophila* Improves Survival to Gram-Negative Bacterial Infections

**DOI:** 10.1371/journal.pone.0004490

**Published:** 2009-02-16

**Authors:** Anthony E. Brown, Janina Baumbach, Peter E. Cook, Petros Ligoxygakis

**Affiliations:** 1 Genetics Unit Department of Biochemistry, University of Oxford, Oxford, United Kingdom; 2 Peter Medawar Building for Pathogen Research, University of Oxford, Oxford, United Kingdom; University of Cambridge, United Kingdom

## Abstract

**Background:**

Primary immunodeficiencies are inborn errors of immunity that lead to life threatening conditions. These predispositions describe human immunity *in natura* and highlight the important function of components of the Toll-IL-1- receptor-nuclear factor kappa B (TIR-NF-κB) pathway. Since the TIR-NF-κB circuit is a conserved component of the host defence in higher animals, genetically tractable models may contribute ideas for clinical interventions.

**Methodology/Principal Findings:**

We used immunodeficient fruit flies (*Drosophila melanogaster*) to address questions pertaining to survival following bacterial infection. We describe here that flies lacking the NF-κB protein Relish, indispensable for countering Gram-negative bacteria, had a greatly improved survival to such infections when subject to dietary short-term starvation (STS) prior to immune challenge. STS induced the release of Nitric Oxide (NO), a potent molecule against pathogens in flies, mice and humans. Administering the NO Synthase-inhibitory arginine analog N-Nitro-L-Arginine-Methyl-Ester (L-NAME) but not its inactive enantiomer D-NAME increased once again sensitivity to infection to levels expected for *relish* mutants. Surprisingly, NO signalling required the NF-κB protein Dif, usually needed for responses against Gram-positive bacteria.

**Conclusions/Significance:**

Our results show that NO release through STS may reflect an evolutionary conserved process. Moreover, STS could be explored to address immune phenotypes related to infection and may offer ways to boost natural immunity.

## Introduction

Disorders in the TIR-NF-κB pathway are primary immunodeficiencies that have been shown to predispose individuals to pneumococcal and to a lesser extend staphylococcal infections, as well as a selective tendency toward herpes simplex virus encephalitis [1], [2], [3]. This pathway has been shown to have remarkable similarities with those used in *Drosophila* immunity and makes the fruit fly a powerful genetically tractable model organism for the study of the first line host defence to infection [reviewed in 4].

In flies, Toll and Imd (for immune deficiency) have been shown to be the major pathways countering infection [reviewed in 5]. Toll signalling culminates in the translocation of the NF-κB homologue Dif to the nucleus following Gram-positive bacterial or fungal challenge [6]. Imd is deployed primarily against Gram-negative bacteria via the NF-κB homologue Relish [7]. This pathway is modulated in larvae by NO [8]. The working hypothesis is that ingestion of bacteria induces NO Synthase (NOS) in the gut. NO released from the gut signals to blood cells, which induce Relish-dependent responses in the fat body (the insect analogue of the liver), the major site of antimicrobial peptide production [8]. According to this model there are two interconnected and sequential phases of the NO-controlled pathway: an NF-κB-independent (blood cells) and an NF-κB-dependent module (fat body). Considering these data, together with the fact that a) NO is a potent antimicrobial agent in a variety of organisms [9], and b) that dietary restriction (DR) activates the endothelial production of NO in mice [10], we investigated whether we could take advantage of the NF-κB-independent phase of NO function to improve the survival of infected Relish-deficient flies if, prior to bacterial challenge, we controlled their access to nutrients.

Our rationale stemmed from the hypothesis that DR-mediated NO production may be an evolutionary conserved process and such a diet regime could be used to boost an immuno-compromised immune system (such as one lacking Relish or more generally a TIR-NF-κB component) by elevating NO levels. The intriguing implication if this hypothesis was correct would be that a DR protocol could be used in humans to manipulate natural immunity and boost host defences without the need for a sophisticated clinical setting.

Our results showed that indeed NO release following a protocol of food restriction is an evolutionary conserved process. Short-term starvation (STS) positively influenced the survival of *relish (rel)* mutant flies following Gram-negative bacterial infection. Bacterial load was markedly decreased in *rel* STS flies compared to their fed counterparts. These phenotypes were reversible when a known NO-inhibitor (L-NAME) was used.

Our results revealed that in wild type flies there was a Relish-dependent positive feedback loop that enhanced NO production following infection, through NOS upregulation. In the absence of Relish, STS stimulated the Toll pathway where Dif activated Cytochrome Oxidase C (CCO), which in turn elevated NO levels without NOS upregulation.

## Results

### STS improves survival of rel-deficient flies to Gram-negative bacteria

**Figure 1:**
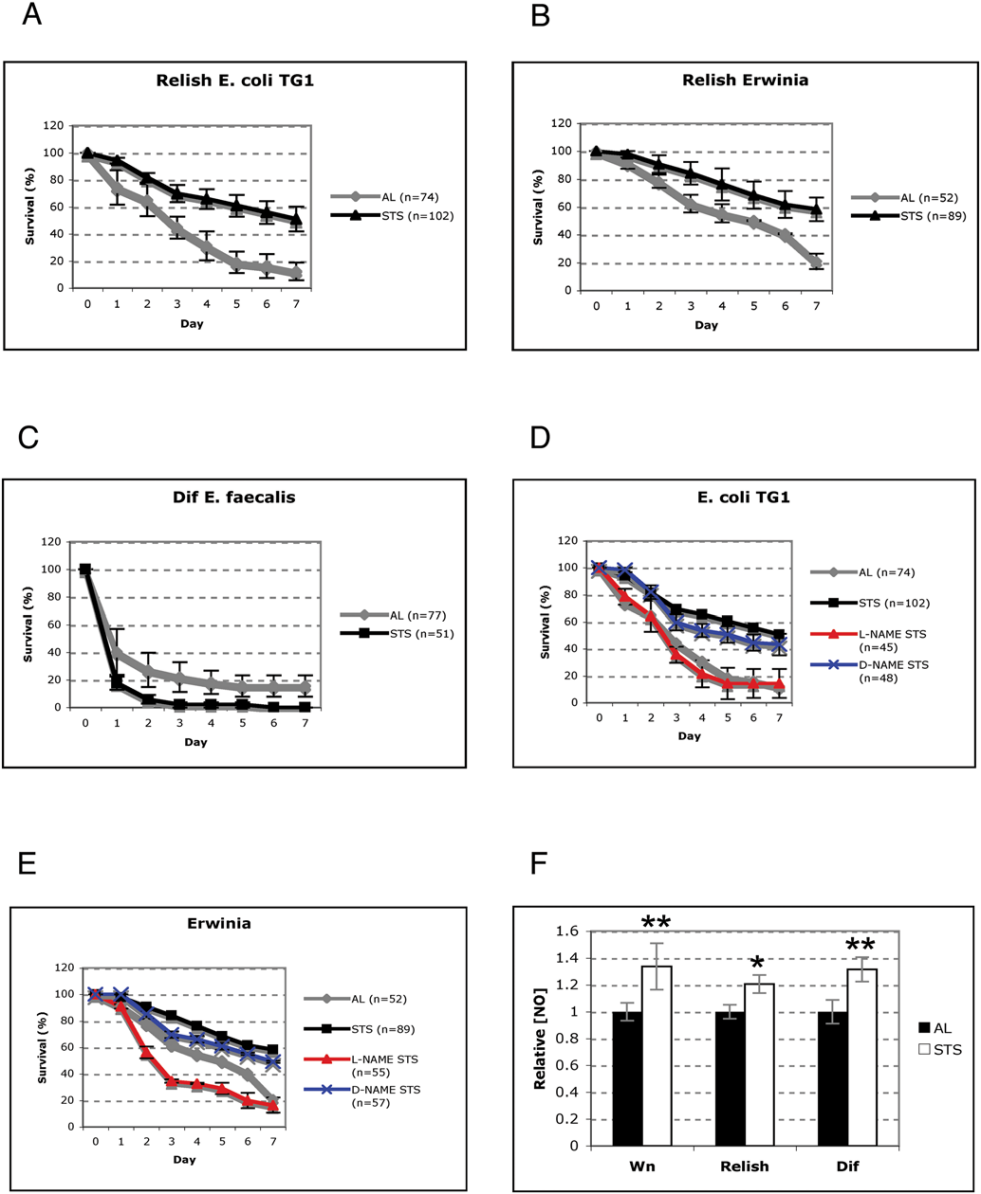
STS enhances Drosophila survival after septic injury. (A) Seven-day survival curve of fed (AL; grey) or 24 hour starved *rel* flies (STS; black line) after infection with *E. coli*. Graphs show mean survival (±standard error [s.e.]) from four independent experiments. (B) Seven-day survival curve of STS *rel* (black line) or AL *rel* flies (grey line) after infection with *Erwinia carotovora*. Graphs show mean survival (±s.e.) from four independent experiments. (C) Seven-day survival of AL *dif* (grey line) or STS *dif* flies (black line) after infection with *Enterococcus faecalis*. Graphs show mean survival (±s.e.) from four independent experiments. (D) Seven-day survival of STS *rel* flies infected with *E. coli*. Newly eclosed flies had either been fed on media supplemented with the NOS inhibitor L-NAME (red line) or its inactive analogue D-NAME (blue line) for 48 hours before the STS regimen was enforced. In either case flies were returned to the L- or D-NAME vial after STS and infection. Graphs show mean survival (±s.e.) from four independent experiments (E) seven-day survival of STS *rel* flies infected with *Erwinia carotovora*. Newly eclosed flies had either been fed on media supplemented with the NOS inhibitor L-NAME (red line) or its inactive enantiomer D-NAME (blue line) for 48 hours before the STS regime was enforced. In either case flies were returned to the L- or D-NAME vial after STS and infection. Graphs show mean survival (±s.e.) of approximately 20 flies from four independent experiments. (F) Quantification of cellular nitric oxide in wild-type (Wn), *rel*, or *dif* flies having had free access to nutrients (AL; black bar) or after STS (white bar). In each case mean STS nitric oxide levels are normalised to the level in AL flies (of 1). Graphs show mean relative NO concentration of 15 male flies from four independent experiments (±s.e.). Asterisk indicates significance value of the result as determined by Student's t-Test (*P = <0.05, **P = <0.01).

In order to determine the effect of food restriction upon NO production in *Drosophila*, we placed *rel* or *dif* flies in nutrient-free agar vials for twenty-four hours. Throughout this period flies had free access to water. We term this short-term starvation (STS) as opposed to dietary restriction (DR) since there was no dilution of the food medium usually applied in DR [reviewed in 11]. Instead, flies were subject to a starvation regime. Furthermore, as it has been estimated that *Drosophila* only consumes one to two microlitres of food per 24 hours [12], our protocol was a short-term restriction of nutrients rather than DR feeding. Two groups of *rel* or *dif* flies were used. Prior to bacterial challenge, one was fed *ad libitum* (AL) while the other was subjected to STS for 24 h. They were then infected with Gram-negative (*rel*) or Gram-positive bacteria (*dif*). After infection flies were transferred to fresh food and surviving individuals counted daily (see materials and methods). STS *rel* flies showed a greatly improved pattern of survival in comparison to AL *rel* flies following infection with *Escherichia coli* (Fig 1A) and *Erwinia carotovora* (Fig 1B). With both pathogens, 80 to 90% of AL *rel* flies died within seven days of infection. STS flies however, improved their survival rate considerably with only 40% of STS *rel* flies dying within the same period. In contrast, STS *dif* flies infected with the Gram-positive bacterium *Enterococcus faecalis* showed similarly high levels of susceptibility to infection as their AL siblings and died rapidly after septic injury (Fig 1C).

In *Drosophila*, NO is active only against Gram-negative bacteria [8]. Since our results also demonstrated that STS specifically countered Gram-negative bacterial infections we pursued our study with *rel* STS and AL flies to investigate whether our results might also be NO-dependent. When both STS and AL *rel* flies were transferred to food containing L-NAME, an inhibitor of NO Synthase (NOS), the two groups exhibited comparable patterns of survival that reflected the susceptibility of *rel* mutant flies to Gram-negative bacteria (Fig 1D, 1E). However, this was not the case when we used the inactive enantiomer D-NAME, indicating specificity in the L-NAME mediated inhibition observed (Fig 1D, 1E). To formally prove that NO release was induced during STS we measured NO levels in STS and AL animals. STS flies had a 20% increase in the levels of NO in comparison to fed animals, regardless of their genotype (Fig 1F). These results indicated that NO induction following food restriction is an evolutionary conserved process [see also 10].

### NO release following STS reduces bacterial load

**Figure 2:**
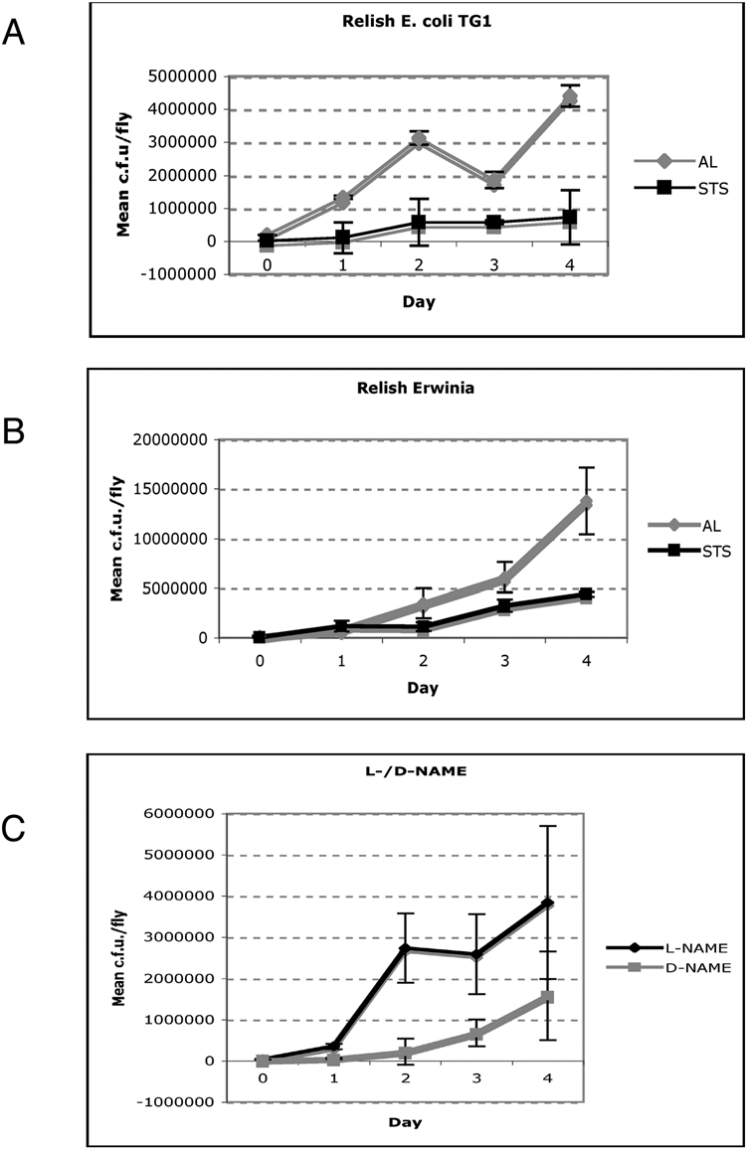
STS results in containment of bacterial proliferation. (A) Mean bacterial density per fly of *rel* flies infected with *E. coli* after being subject to AL (grey line) or STS (black line) feeding regimens. Data show mean colony forming units (c.f.u.) per fly (±s.e.) from four independent experiments. (B) Mean bacterial load of *rel* flies infected with *Erwinia* after AL (grey line) or STS (black line) feeding regimens. Data show mean c.f.u. per fly (±s.e.) from four independent experiments. (C) Mean bacterial load of STS *rel* flies infected with *E. coli* and cultured on media supplemented with the NOS inhibitor L-NAME (black line) or its inactive enantiomer D-NAME (grey line). Data show mean c.f.u. per fly (±s.e.) from four independent experiments.

Production of NO in mice and humans can be directly correlated with the ability of the host to limit microbial proliferation [13] We observed that improvement in disease susceptibility of STS *rel* flies also correlated with containment of infection. This was determined by assaying the proliferation of bacteria in each fly (CFU/fly) (Fig. 2). For this purpose we used a CFP-*E. coli* strain or a YFP-*Erwinia carotovora* strain (see materials and methods). During 96 h of observation the mean bacterial load was constrained at roughly the same level in STS *rel* animals during both infections (Fig 2A, 2B). In contrast, proliferation was seen in AL *rel* individuals where the initial mean bacterial load increased 5-fold over the 96 hour period (Fig 2A, 2B). This difference in bacterial proliferation was not observed however, in food containing L-NAME (Fig 2C). Again, the inactive enantiomer D-NAME had no effect in influencing bacterial load (Fig 2C). These results demonstrated that NO was at the centre of infection containment.

### NO acts both as a signalling molecule and as an antimicrobial agent

**Figure 3:**
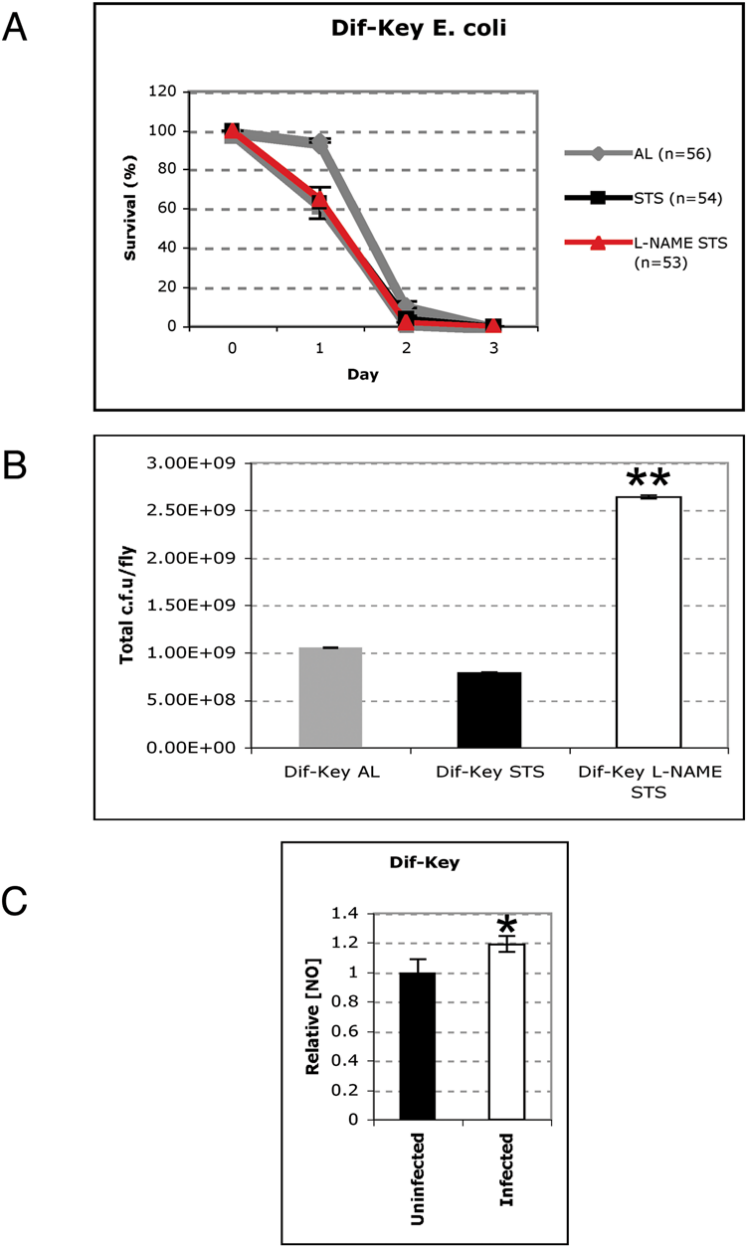
Enhanced survival after STS is dependent upon NF-κB signaling. (A) Survival curve of *E. coli*-infected *dif-key* flies after AL (grey line), STS (black line) or STS and L-NAME-treatment (red line). Graphs show mean survival (±s.e.) from three independent experiments. (B) Total mean bacterial density of *dif-key* flies after infection with *E. coli* after AL (grey bars), STS (black bars) or treatment with L-NAME and STS (white bars). The total mean c.f.u. per fly (±s.e.) from three individual experiments are shown. Double asterisk (**) indicates a statistically significant difference in value from all other values (Student's t-Test; *P* = <0.01). (C) Quantification of cellular nitric oxide in *E. coli*-infected *dif*-key flies (white bars). Graphs show relative NO concentration (to uninfected flies [black bars]) of 15 male flies from three independent experiments (±s.e.). Asterisk (*) indicates statistically significant difference in the mean value in comparison to the other mean values presented in the graph as determined by Student's t-Test (*P* = <0.05).

Abrogation of inducible NO activity produces dramatic increases in microbial burden perpetuating the idea that NO has direct antimicrobial activity [13]. However, in *Drosophila* the working model is that NO is functioning as a signalling molecule dependent on the Imd-Relish pathway [8]. Surprisingly, our results suggested that the effects of NO-induction we observed after starvation were independent of Relish (Fig 1F). Hence there was a case for a direct antimicrobial role. To distinguish between an NF-κB- and a direct NO-mediated effect we used *dif-key* flies [14], a strain double mutant for *dif* and *kenny* (*key*) the IKKγ/NEMO component homologue of the fruit fly's IκB-Kinase complex, which is essential for Relish activation [15]. STS *dif-key* flies did not show an improved survival pattern compared to AL *dif-key* mutants (Fig 3A). Moreover, no difference was observed between the mean bacterial loads of AL and STS *dif-key* flies (Fig 3B) However, the STS *dif-key* flies were still exhibiting a moderate containment of the bacterial load over time compared to L-NAME-treated STS *dif-key* flies (Fig 3B). This suggested that one component of NO function was independent of NF-κB released upon infection (Fig. 3C), and that this component could be acting directly as an antimicrobial agent. Nevertheless, the differences in survival between STS rel and STS *dif-key* flies indicated the presence of a major NF-κB-related component in which (in the absence of Relish) Dif itself was mediating NO action.

**Figure 4:**
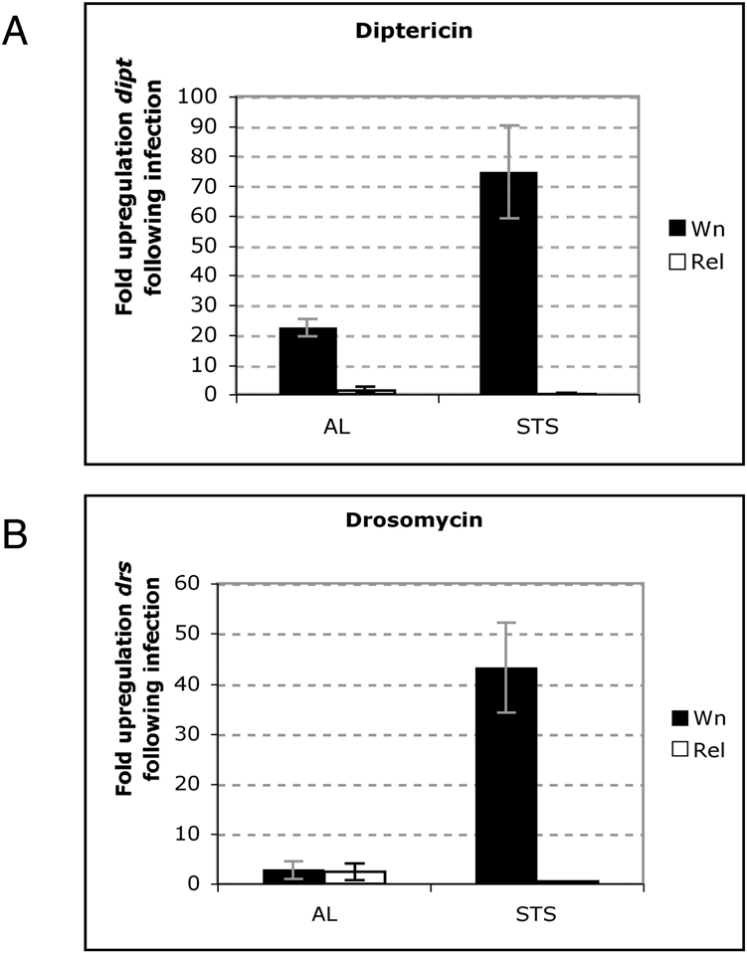
Antimicrobial peptide expression in flies subject to STS. *Diptericin* (A) and *Drosomycin* (B) expression in AL or STS wild-type (Wn; black bars) and *rel* flies (white bars) after infection with *E. coli*. Data from three independent experiments show the fold change (±s.e.) in AMP expression after *E. coli* infection, normalised to the internal reference gene *Rp49*.

How then might Dif be mediating NO action? NO signalling induces NF-κB-dependent production of the antimicrobial peptides (AMPs) diptericin and drosomycin [8]. To determine whether Dif was directing AMP expression through NO (thus improving survival of STS rel flies), we measured AMP gene expression using quantitative real-time PCR. As expected the AMP gene *diptericin (dipt)*, a read-out for Imd pathway activation following Gram-negative bacterial challenge [7], was not induced after *E. coli* infection of AL *rel* flies (Fig 4A). This was also the case for STS *rel* flies, indicating an absolute dependence of *dipt* expression on Relish (Fig 4A). Similarly, *drosomycin* (*drs*) was not upregulated in *rel* flies following Gram-negative challenge (Fig 4B). However, *drs* expression was increased approximately 70-fold following STS (and infection) in wild type flies suggesting that NO can direct Relish-mediated induction of *drs* (Fig 4B). Together these results indicated that the amelioration of STS *rel* flies survival was not dependent upon AMPs, since loss of Relish abolished AMP induction.

### STS induces a positive feedback loop for NO release

**Figure 5:**
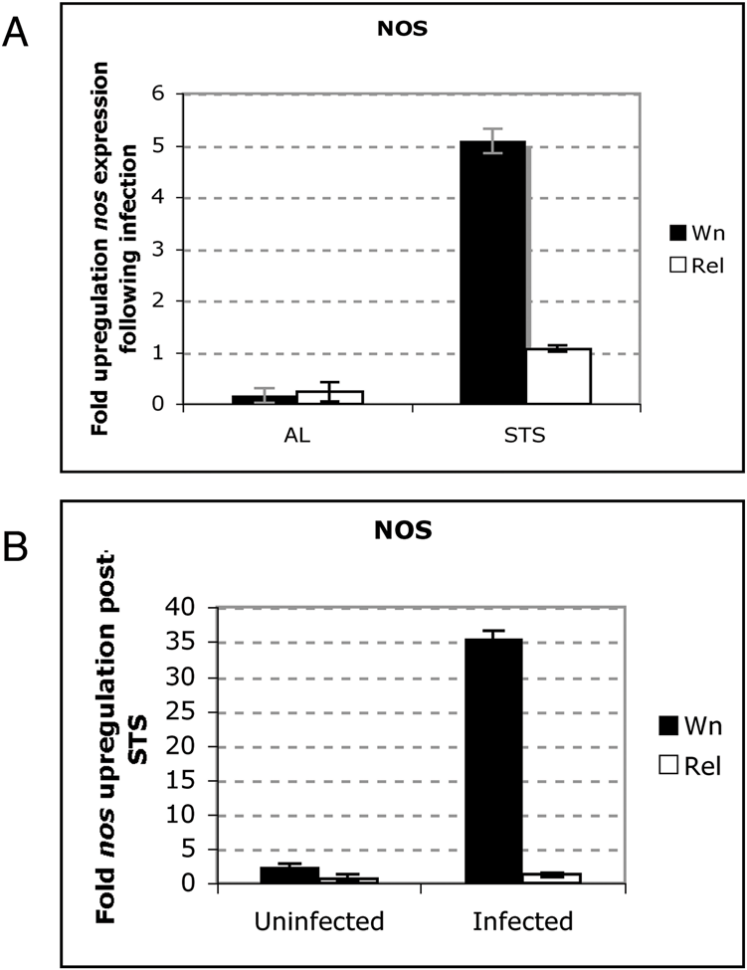
NOS is upregulated by STS and infection. (A) Fold-change in *NOS* expression in AL or STS wild-type (black bars) or *rel* (white bars) flies following *E. coli* infection. (B) Fold-change in *NOS* expression in uninfected or *E. coli*-infected STS wild-type (black bars) or *rel* (white bars) flies. In all cases mean expression levels (±s.e.) from three independent experiments are shown.

We next sought to determine the aspects of NO expression and/or signalling that promoted STS *rel* survival. To this end we monitored transcription of the *Nitric Oxide Synthase* (*NOS*) gene. STS induced the upregulation of *NOS* in both wild type and *rel* flies (Fig. 5A). In an effort to determine whether STS-mediated upregulation was dependent upon NF-κB, we quantified *NOS* expression in uninfected and *E. coli* infected STS flies. While *NOS* was upregulated 1- to 2-fold following STS (Fig. 5B) (that correlated with an approximately 0.2-fold increase in total cellular NO concentration [Fig. 1F]), infection of these STS flies with *E coli* led to a 35-fold increase in *NOS* expression in wild-type flies that was not observed in *rel* flies (Fig. 5B). This suggested that while NO synthesis after STS (and subsequently, infection) had an NF-κB-independent component (the fact that we observed increased NO in both *rel* and *dif-key* flies showed this), Relish could specifically enhance NO signalling via *NOS* upregulation. In wild type STS flies this would constitute a positive feedback loop: infection would sequentially induce NO production, Relish activation [8] and finally, NOS up-regulation, which would lead to more NO thus completing the circle. Nevertheless, our results also indicated that STS primed the initiation of such a loop that was evident following infection of STS *rel* but not of AL *rel* flies (Fig 5A white bars), with Dif presumably fulfilling the role of Relish.

### STS induces mitochondrial biogenesis

**Figure 6:**
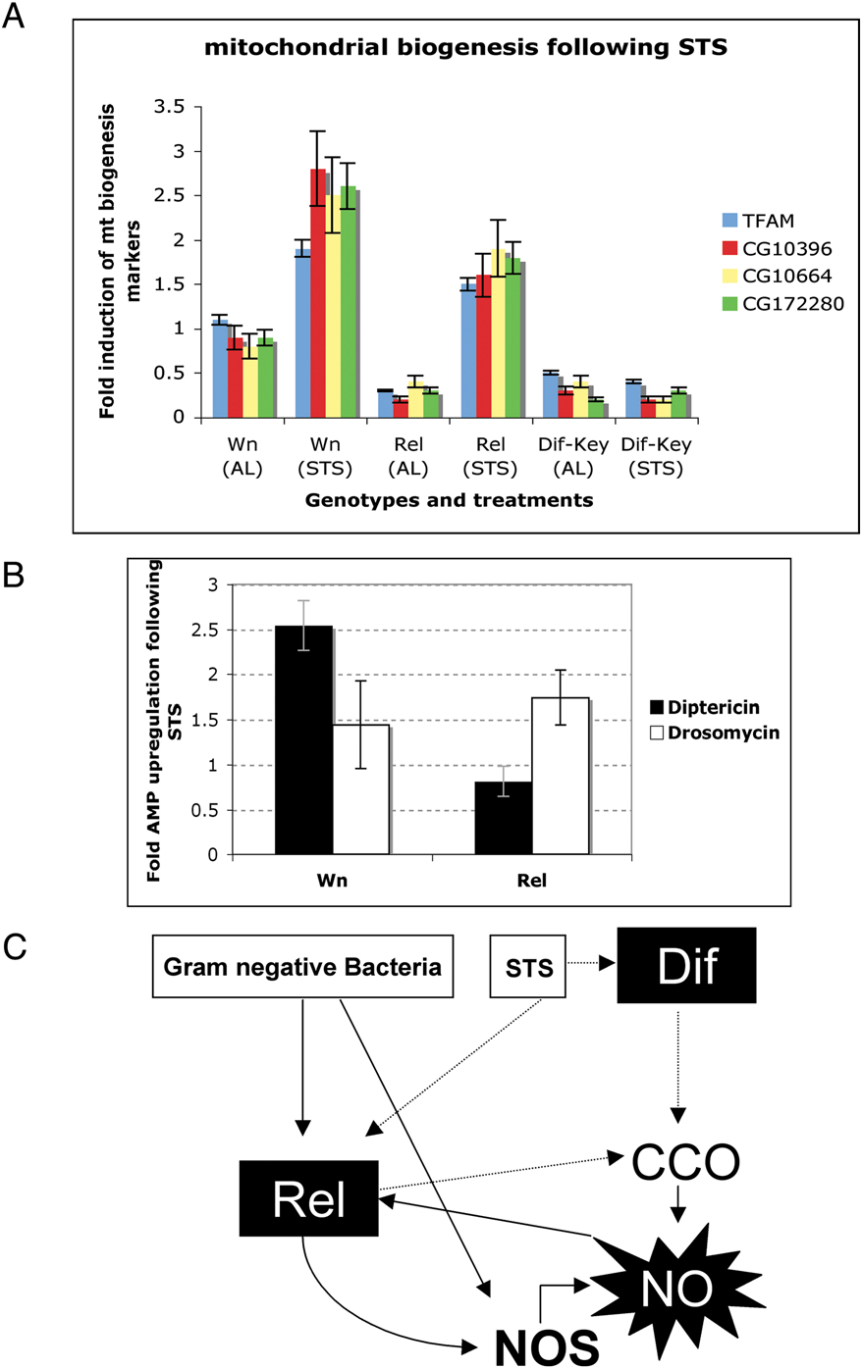
STS induces mitochondrial biogenesis. (A) Fold change in the expression of genes used as markers for mitochondrial biogenesis following STS compared to AL conditions. Measurements by real-time quantitative PCR were corrected with *rp49* expression as in [26]. The genes used were *tfam*, master regulator of mitochondrial biogenesis; CG13096 and CG10664, *cox4*; CG17280, *cox6A*. Note that the induction observed was dependent on NF-κB since it was not observed when both Toll-Dif and the Imd-Relish pathway were deficient (Dif-Key) (B) AMP gene expression is induced upon starvation. Fold-change in *Diptericin* (black bars) or *Drosomycin* (white bars) expression in wild type (*Wn*) or *rel* flies following STS. Induction of AMPs implies that NF-κB signalling is robustly activated following STS. In all cases in (A) and (B) mean expression levels (±s.e.) from three independent experiments are shown. (C) Putative mode of regulation of NO expression in *Drosophila*. NO is synthesised via overlapping and interconnected pathways. The main component of this mode is achieved through the direct upregulation of NOS mediated by the NF-κB protein Relish after infection by Gram-negative bacteria. NO can then upregulate the IMD pathway in a positive feedback loop. A second (minor) component of this would be the direct (NF-κB-independent) production of NO as an antimicrobial agent against Gram-negative bacteria. Finally, STS-mediated NO expression is catalysed by two-independent means. Principally through the up-regulation of Relish, leading to NOS upregulation as described above, or secondly, in a NOS-independent fashion. In this case starvation-induced NF-κB (either Relish and/or Dif) upregulation leads to CCO upregulation and NOS-independent NO production.

We next sought to determine how STS might regulate NO expression through Dif. NO generated after DR increased mitochondrial biogenesis and enhanced respiration and ATP content in various mammalian cells [10]. To investigate whether a similar phenomenon occurred in our system we monitored transcription of different mitochondrial markers (Fig 6A). Transcript levels of the *Drosophila* homologue of the mitochondrial transcription factor A (Tfam; master regulator of mitochondrial biogenesis) [16] and the putative *cytochrome oxidase c* (CCO) subunits CG10396 (COX IV), CG10664 (COX IV) and CG17280 (COX6A) were monitored by RT-PCR. We observed upregulation of all these mitochondrial markers when subject to STS (Fig 6A). Moreover, we observed an upregulation of *dipt* and *drs* following STS (Fig. 6B) that indicated STS may also activate NF-κB as observed previously [17].

Mitochondria from yeast, rat liver, and plants are capable of NOS-independent NO synthesis [reviewed in 16] when subject to hypoxia and this reaction is catalysed by CCO [18]. Since CCO has been shown to be under *Dif* and *Relish* control [Fig 6A] and NF-κB signalling was activated by STS (as measured by AMP induction shown in Fig. 6B), this mechanism may present a way of augmenting intracellular NO without increasing the transcription levels of NOS, as would be the case in the absence of Relish (Fig 5B). This in turn could lead to a positive feedback loop with NO accumulation directing NF-κB-mediated signalling towards CCO upregulation and AMP production (Fig 6C; see below). This may be occurring in STS *rel* flies, augmenting their cellular NO content via Dif in the absence of any significant change in *NOS* expression, hence ameliorating the outlook for their survival when compared to *dif-key* flies.

## Discussion

Involved in many responses to various exogenous and endogenous signals are the evolutionary conserved NF-κB transcription factors. Recent studies have shown that in mice, DR acts as a trigger for NO activation [10]. We show here that this procedure is conserved in *Drosophila*. Moreover, it can be used to ameliorate the outlook of Relish immunodeficient flies following Gram-negative infection. Surprisingly our data show that in the absence of Relish, which is the main mediator of NO effects in wild type flies [8], the Toll-specific NF-κB regulator Dif, is recruited to direct NO synthesis. To our knowledge, this is the first direct *in vivo* evidence of a role for Dif in promoting NO function.

Experiments presented here as well as those of a recent study, have shown that STS induces the Toll pathway [17]; [Fig 6B], which in turn can activate CCO [this study, see also 10]. In the absence of Relish, STS may be inducing Dif-directed CCO-mediated NO production. This could lead to mitochondrial biogenesis as judged by the up-regulation of several related markers (Fig 6A). Our results suggest that the link between food restriction-mediated production of NO and mitochondrial biogenesis is evolutionary conserved. Finally, in wild type flies infection leads to Relish-mediated enhancement of *NOS* expression, with the NO produced signalling to the IMD pathway to augment AMP expression, that in turn enhances NOS expression and NO production (Fig 6C).

In mice, intra-hepatic synthesis of NO through the expression of a NOS-2 transgene, leads to protection from liver inflammatory injury [19]. Our results strongly parallel this and indicate that the protective mechanism in flies is NF-κB dependent. However, NO was not strictly a signalling molecule but acted also directly as an antimicrobial agent (Fig. 2). Recent results concerning primary immunodeficiencies in humans indicate overlapping host defences *in natura* [1]. The overlapping action of NO and AMPs observed in *Drosophila* underlie the evolutionary implications of such a notion.

Manipulating NO synthesis through diet may have considerable consequences for developing strategies to protect immunodeficient individuals and future studies with a graded nutritional supply [20] should reveal, which parts of the fly diet may be restricted in order to activate NO in *rel* flies. It is highly significant to this study that nutritional status has also been shown to modify immune outcome in humans by preferentially altering the differentiation of CD4^+^ naive T helper (Th) 0 [21]. Differentiated Th1 cells primarily stimulate cell-mediated immunity against intracellular pathogens by activating macrophages and CD8^+^ cytotoxic T lymphocytes that leads to the production of gamma interferon (IFN-γ) [reviewed in 22]. Th2 cells, on the other hand, favour the B-cell-dependent humoral immune response against extracellular pathogens and the production of interleukin 4 (IL-4) [23]. When healthy human volunteers were starved overnight, levels of IL-4 increased. Food intake on the other hand resulted in increased levels of IFN-γ [21]. The mechanism for the differential modification of the Th1-Th2 balance in response to calorific input remains enigmatic, however, it is tempting to speculate it might also involve NO. In mammals NO negatively influences Th1 cell development by limiting IL-12 production from macrophages [24], which may therefore lead to the preferential differentiation of Th2 cells. The results of our study suggest it may be appropriate to trial an STS-based protocol in immunocompromised humans.

In conclusion, we have presented evidence of a protocol for the improvement of survival as well as the containment of bacterial load following infection with Gram-negative bacteria in immunocompromised *Drosophila*. This non-invasive procedure relied on the potential of priming natural elements of the immune system with the ability to respond better to infection. This protocol involved short-term starvation (STS) prior to septic injury. STS mediated the release of NO principally through an NF-κB-dependent mechanism although there was a minor component of NO that was independent of NF-κB. More experiments are needed to fully understand this latter mechanism.

## Materials and Methods

### Fly stocks

The following stocks were used in this study: *Wn*, an isogenised wild type strain, which was the strain used as a background to generate the *rel^20E^* mutant [7], *rel^20E^* [7], *dif* [6] and *dif-key* [14].

### Fly culture and *ad libitum* feeding

Flies were raised in standard vials on food (2% agar, 10% treacle, 10% wholemeal flour, 25 mM Methyl-4-hydroxybenzoate, 0.5% propionic acid) at 25°C.

### Short term starvation (STS)

Day old flies were transferred to nutrient-free vial (2% agar, 25 mM Methyl-4-hydroxybenzoate, 0.5% propionic acid) and incubated at 25°C for 24 h.

### NOS inhibition

Newly eclosed flies that had been raised on standard food were transferred to standard food supplemented with 100 mM L-NAME or D-NAME for 48 h before being subject to AL or STS feeding regimens. In each case, L- or D-NAME was included with the specific feeding regimen used. After infection flies were returned to L- or D-NAME-containing food.

### Bacterial strains and infection experiments

The following bacteria were used in this study: Gram-negative: *Escherichia coli* TG1; *E. coli* TG1:ECFP (an *E. coli* strain expressing cyan fluorescent protein developed by transforming it with pECFP-1 [Clontech]); *Erwinia carotova*; *Erwinia*:YFP (Erwinia expressing yellow fluorescent protein developed by transforming it with pEYFP-1 [Clontech]) *E. coli* JM109 and *E. coli* JM109:pQE60-G57 (JM109 that expresses a C-terminal 6×His fusion to the *KatN* gene under control of isopropylthio-*β*-D-galactoside (IPTG)-inducible promoter [Robbe-Saule]). Gram positive: *Enterococcus faecalis*. Bacterial immune challenge was performed as described previously [6].

### Bacterial proliferation assay

The cuticles of 3 male flies were sterilised by brief immersion in 70% ethanol before being homogenised in 100 µl Luria Bertani (LB) broth. 10 µl of the homogenate was added to 990 µl LB broth (with further dilution if necessary) and 10 µl of this plated on LB agar plates supplemented with 60 µg ml-1 carbenicillin and 250 ng ml-1amphotericin B (Fungizone; Invitrogen).

### Nitric Oxide Assay

15 male flies (heads removed) were homogenised in 250 µl PBS supplemented with 1 mM Na_2_O_4_S_4_. Homogenates were sonicated debris collected by centrifugation at 5,000×g. High molecular weight proteins, which would otherwise interfere with the NO assay were removed from the lysates with Ultrafree Biomax 10 kDa columns (Millipore) by centrifugation at 11,000 rpm for 15 min. Total NO in the sample was quantified using the Endogen Total Nitric Oxide Assay Kit (Pierce) according to the manufacturer's protocol.

### Quantitative Reverse Transcriptase PCR (qRT-PCR)

Following 24 h STS (see above) total RNAs were extracted from either eight male or five female flies using TRIzol (Invitrogen). Total RNA was treated with DNAse RQ1 (Promega) and gene expression levels of Diptericin, Drosomycin and NOS quantified from *Wn* and *rel* AL and STS flies using a one-step quantitative RT-PCR (qRT-PCR). One-step qRT-PCR was performed using 1 µg of total RNA with the SensiMix one-step kit (Quantace) according to the manufacturer's SYBR Green I protocols. Reactions were run on a DNA Engine thermocycler (MJ Research) with Chromo4 real-time PCR detection system (Bio-Rad) using the following cycling conditions: 42°C for 30 mins; 95°C for 10 mins; then 45 cycles of 95°C for 20 s, 57°C for 20 s, 72°C for 30 s, fluorescence acquisition; then a melt curve analysis with fluorescence measurements taken at 0.3°C steps from 65°C to 95°C. Triplicate reactions from two independent experiments were run for each primer set across all templates. The cycle threshold (C_t_) value was determined and gene expression levels of target genes calculated, relative to the internal *Drosophila* reference gene *Rp49*, using the ΔΔC_t_ method [25]. The following primers were used for qRT-PCR: *Diptericin*:- 5′-CCGCAGTACCCACTCAATCA-3′and R: 5′-ACTTTCCAGCTCGGTTCTGA-3′; *Drosomycin*: 5′-CTCTTCGCTGTCCTGATGCT-3′and R: 5′-CGCACCAGCACTTCAGACT-3′; *NOS*: F: 5′-AGCAACAGAAGGCACAGACA-3′and R: 5′-AGGCGATGCTGTGGAGATAC-3′; *Rp49*:- F: 5′-TCCTACCAGCTTCAAGATGAC-3′and R: CACGTTGTGCACCAGGAACT-3′; *tfam*:- F: 5′-TCCCTACTTTCGCTTCATGC-3′ and R: 5′-TCGCTCCTCCACGTAGATTT-3′; *CG10396*: F: 5′-AGGTCGTTGGTGATGGAATC-3′and R: 5′-CATTCGGGTGTAATGTGCTG-3′; CG10664: F: 5′-ACCAACGAGATCAACGCTCT-3′ and R: 5′-GCGCAGAAGAGGAGTGAAAC-3′; *CG17280*: F: 5′-TCTGGTGGCTACAAGGTGTG-3′and R: TTGTGGAACAGGCTCTTCTG-3′.

## References

[1] Casanova JL, Abel L (2007). Primary immunodeficiencies: a field in its infancy.. Science.

[2] Picard C (2003). Pyogenic bacterial infections in humans with IRAK-4 deficiency.. Science.

[3] Zhang (2007). TLR3 deficiency in patients with herpes simplex encephalitis.. Science.

[4] Akira S, Uematsu S, Takeuchi O (2006). Pathogen recognition in innate immunity.. Cell.

[5] Wang L, Ligoxygakis P (2006). Pathogen sensing and signalling in Drosophila immune response.. Immunobiol.

[6] Rutchmann S, Jung AC, Hetru C, Reichhart JM, Hoffmann JA, Ferrandon D (2000). The Rel protein DIF mediates the antifungal but not antibacterial host defence in Drosophila.. Immunity.

[7] Hedengren M, Asling B, Dushay MS, Ando I, Ekengren S, Wihlborg M, Hultmark D (1999). Mol Cell.

[8] Foley E, O'Farrell PH (2002). Nitric oxide contributes to induction of innate immune responses to Gram-negative bacteria in Drosophila.. Genes Dev.

[9] Bogdan C (2001). Nitric oxide and the immune response.. Nat Immunol.

[10] Nisoli (2005). Calorie restriction promotes mitochondrial biogenesis by inducing the expression of eNOS.. Science.

[11] Piper MDW, Partridge L (2007). Dietary restriction in Drosophila: delayed aging or experimental artefact?. PLoS Genet.

[12] Carvalho GB, Kapahi P, Benzer S (2005). Compensatory ingestion upon dietary restriction in Drosophila.. Nat Methods.

[13] Fang FC (1997). Mechanisms of Nitric Oxide-related antimicrobial activity.. J Clin Invest.

[14] Rutschmann S, Kilinc A, Ferrandon D (2002). Cutting edge: the toll pathway is required for resistance to gram-positive bacterial infections in Drosophila.. J Immunol.

[15] Rutschmann S, Jung AC, Zhou R, Silverman N, Hoffmann JA, Ferrandon D (2000). Role of Drosophila IKK gamma in a toll-independent antibacterial immune response.. Nat Immunol.

[16] Kelly DP, Scarpulla RC (2004). Transcriptional regulatory circuits controlling mitochondrial biogenesis and function.. Genes Dev.

[17] Wu J, Randle KE, Wu LP (2007). Ird1 is a Vps15 homologue important for antibacterial immune responses in Drosophila.. Cell Microbiol.

[18] Castello PR, David PS, McClure T, Crook Z, Poyton RO (2006). Mitochondrial cytochrome oxidase produces nitric oxide under hypoxic conditions: implications for oxygen sensing and hypoxic signaling in eukaryotes.. Cell Metab.

[19] Mojena M (2001). Protection by nitric oxide against liver inflammatory injury in animals carrying a nitric oxide synthase-2 transgene.. FASEB J.

[20] Magwere T, Chapman T, Partridge L (2004). Sex differences in the effect of dietary restriction on life span and mortality rates in female and male *Drosophila melanogaster*.. J Gerontol.

[21] van den Brink GR (2002). Feed a cold starve a fever?. Clin Diagn Lab Immunol.

[22] Coffman CL (2007). Origins of the T(H)1-T(H)2 model:. Nat Immunol.

[23] Cher DJ, Mosmann TR (1987). Two types of murine helper T cell clone. II. Delayed-type hypersensitivity is mediated by TH1 clones.. J Immunol.

[24] Huang FP, Niedbala W, Wei XQ, Xu D, Feng GJ, Robinson JH, Lam C, Liew FY (1998). Nitric oxide regulates Th1 cell development through the inhibition of IL-12 synthesis by macrophages.. Eur J Immunol.

[25] Livak KJ, Schmittgen TD (2001). Analysis of relative gene expression data using real-time quantitative PCR and the 2[-Delta Delta C(t)] method.. Methods.

